# Stable isotope and fatty acid variation of a planktivorous fish among and within large lakes

**DOI:** 10.1371/journal.pone.0304089

**Published:** 2024-07-22

**Authors:** Tomas O. Höök, Nicholas I. Kalejs, Thomas Axenrot, Martin Ogonowski, Alfred Sandström

**Affiliations:** 1 Department of Forestry and Natural Resources, Purdue University, West Lafayette, IN, United States of America; 2 Illinois-Indiana College Sea Grant Program, West Lafayette, IN, United States of America; 3 Department of Aquatic Resources, Institute of Freshwater Research, Swedish University of Agricultural Sciences, Drottningholm, Sweden; 4 Maine Department of Inland Fisheries and Wildlife, Augusta, ME, United States of America; Universidad de Cadiz Facultad de Ciencias del Mar y Ambientales, SPAIN

## Abstract

Aquatic food webs are spatially complex, potentially contributing to intraspecific variability in production pathway reliance of intermediate trophic level consumers. Variation in trophic reliance may be described by well-established trophic indicators, like stable isotope ratios (δ^13^C, δ^15^N), along with emerging trophic indicators, such as fatty acid composition. We evaluated stable isotope ratios and fatty acid profiles of European smelt (*Osmerus eperlanus*) among and within distinct regions of three large Swedish lakes (Hjälmaren, Mälaren, Vättern) which differed in trophic status. We expected that smelts in more oligotrophic lakes and regions would be characterized by distinct stable isotope signatures and fatty acid profiles, with particularly high polyunsaturated fatty acid (PUFA) relative levels. However, we acknowledge that frequent movement of smelts among regions may serve to spatially integrate their diet and lead to limited within-lake variation in stable isotope ratios and fatty acid composition. As expected, in comparison with more productive lakes (i.e., Hjälmaren and Mälaren), smelts from ultra-oligotrophic Vättern were characterized by low δ^15^N, high δ^13^C and high percent of a dominant PUFA, docosahexaenoic acid (DHA). Smelts from different regions of the morphometrically complex Mälaren displayed differential stable isotope ratios and fatty acid relative concentrations, which were consistent with within-lake differences in productivity and water residence times, suggesting that smelts in this lake forage locally within distinct regions. Finally, at the individual smelt level there were particularly strong and consistent associations between a well-established trophic indicator (δ^13^C) and percent DHA, suggesting that the relative concentration of this fatty acid may be a useful additional trophic indicator for smelt.

## Introduction

Aquatic food webs are often spatially complex, with various consumers potentially displaying high intraspecific variation in trophic reliance. Various conceptual models have been developed to represent food web spatial structure (e.g., [1, 2]) and generally suggest that lower order consumers rely on local energy channels (specialize) while top trophic consumers spatially integrate energy channels through broad movements and relatively diverse diets. Spatial food web patterns of intermediate trophic level consumers may be less consistent, potentially reflecting broad trophic integration (through extensive movements and flexible foraging) or local specialization. In lakes, spatially distinct production pathways (e.g., allocthonous, littoral, offshore pelagic, offshore demersal) will differentially support components of the food web. Moreover, in large lake systems, food web spatial complexity may be further enhanced as a) large distances and physical features (e.g., multiple basins in large lakes) may serve to separate food web components and b) environmental conditions (e.g., local riverine inputs, benthic substrate, upwelling zones, and water residence) may vary spatially across broad distances. Thus, in large lakes, intraspecific regional trophic variation may be relatively common among intermediate trophic level consumers, including invertivorous fishes (e.g., [3–6]).

Various measures allow for indexing of intraspecific trophic variation across space, including stomach content analyses, fatty acid (FA) quantification and stable isotope ratios. The latter two measures are particularly insightful as they provide a temporal integration of trophic reliance across time scales from weeks to months. Moreover, a wealth of past studies provide *a priori* expectations of how consumer stable isotope ratios and fatty acid compositions may vary intraspecifically among and within systems. Stable isotopes ratios of carbon and nitrogen (δ^13^C and δ^15^N) have been widely used to describe trophic connections within food webs. In addition, these markers can potentially reveal habitat utilization history (e.g., [7]) and reflect differences in allocthonous input and system trophic status (e.g., [8, 9]). Carbon and nitrogen stable isotopes are incorporated into consumer tissue based on relative concentrations in the diet, albeit not in direct proportion to diet concentrations. Tissue turnover rates largely determine what temporal scale of past diet that stable isotope composition reflects. For example, in slower growing consumers, such as fish, stable isotope ratios of muscle tissues may reflect diets over several months (e.g., [10, 11]). If different habitats or regions contain prey of sufficiently distinct δ^13^C and δ^15^N values, then consumers feeding consistently in these different locations would be expected to express distinct isotope ratios (e.g., [3, 12]), whereas more mobile consumers would express spatially integrated isotopic ratios [2, 13].

Again, while consumer δ^13^C and δ^15^N values have been commonly used to interpret food web connections within a system, baseline (primary producer or primary consumer) differences in δ^13^C and δ^15^N values among or within systems may exceed differences attributable to diet variation. Aquatic producer δ^15^N values are related to various processes including differential taxa-specific incorporation of ^15^N and ^14^N through nitrogen fixation, nitrification and denitrification, as well as different sources of allochthonous nitrogen, some of which may be relatively depleted in ^15^N (common among synthetic fertilizers; [14]) and others may be relatively enriched in ^15^N (more common among organic fertilizers; [14]). However, the relative depletion and enrichment of ^15^N in different fertilizers will vary among sources and systems. The number of processes and factors that influence producer δ^15^N values may seem to confound generalizable patterns. However, several studies have documented elevated δ^15^N values in primary producers, particulate organic matter (POM), sediments and consumers from systems and locations that were more eutrophic and more directly influenced by tributary and anthropogenic loading [8, 15–17]. In addition to such strong effects of N loading source, δ^15^N of POM and primary consumers has been documented to increase from small to large lakes [8, 18]. In contrast, greater water residence time, which is common in larger lakes, along with low catchment to lake surface area ratio would be expected to lead to greater relative contribution of over-lake deposition of nitrogen and hence lower δ^15^N. In total, modest differences in lake size will affect baseline δ^15^N values, but are unlikely to overwhelm the influence of N loading source on baseline δ^15^N values.

Compared to δ^15^N, the relationship between system productivity and consumer δ^13^C values is less well studied and not as consistent across various types of aquatic systems. However, most studies in lacustrine systems indicate that producer [19] and consumer [7, 16, 20, 21] δ^13^C values are indicative of ^13^C depletion at sites that are: a) eutrophic and b) highly influenced by allochthonous inputs and riverine discharges. In less eutrophic lakes with higher water clarity, benthic reliance may be greater [22] and contribute to higher consumer δ^13^C values.

In aquatic systems, higher trophic level consumers are not able to synthesize many fatty acids *de novo*, and instead are largely dependent on obtaining certain essential FAs (e.g., 20:5n-3, eicosapentaenoic acid, EPA and 22:6n-3, docosahexaenoic acid, DHA) from their diet [23]. That said, fish consumers do have abilities to biosynthesize saturated fatty acids and desaturate them to form some unsaturated fatty acids [24]. In addition, fish will preferentially retain certain fatty acids obtained through diet (e.g., [25, 26]). Thus, FA composition of consumers is not expected to be proportional to FA composition of their prey. However, FA composition of consumers is expected to be strongly influenced by FAs consumed and several studies have demonstrated a strong effect of diet on fish consumer fatty acid composition (e.g., [27, 28]).

Primary producers differ in terms of their synthesis and relative concentrations of different fatty acids [29, 30]. Thereby, systems with distinct primary producer communities may be characterized by differences in FA composition among consumers; e.g., i) relatively high concentrations of EPA associated with diatoms and euglenoids, ii) high concentrations of DHA associated and dinoflagellates and chrysophytes, and iii) low levels of both DHA and EPA associated with chlororphytes and cyanobacteria [30, 31]. In particular, producer effects on FA availability have manifested in differences in consumer FA profiles along system productivity continuums [31–33] and among systems characterized by different types and magnitudes of allochthonous inputs (e.g., [33, 34]). For example, it is well-established that certain types of primary producers dominate in more eutrophic (e.g., cyanobacteria) compared to more oligotrophic systems (e.g., diatoms). As a consequence, consumers in more oligotrophic systems are often characterized by higher concentrations of polyunsaturated fatty acids (PUFAs) and potentially high concentrations of specific FAs such as EPA and DHA [17, 33, 35]. In addition, fatty acid composition and indices differ among benthic, pelagic and terrestrial primary producers and consumers. For example, the ratio of ω3 to ω6 PUFAs (ω3:ω6) of consumers has been related to dominant energetic pathways, potentially allowing for differentiation among terrestrial, benthic and pelagic pathways [36–38]. In general, lower ω3:ω6 tends to be related to greater reliance on terrestrial pathways [e.g., 39]. However [30], suggested that ω3:ω6 patterns are inconsistent among aquatic and terrestrial producers and hence suggested caution in using this index to assign dominant production sources. Nonetheless, differences in FA composition directly influence the nutritional value of prey for higher trophic level predators, potentially including humans who rely on fish as an important source of PUFAs [33].

Consumer fatty acid content may also vary intraspecifically within systems. If trophic status or allochthonous influences vary spatially in a system, these factors can contribute to spatial differences in primary producer communities and in the FA composition of consumers. For example, in Lake Michigan, USA, several species of fishes feeding on invertebrates are characterized by intraspecific differences in FA profiles across geographic regions [3–5]. Such differences are most plausible to arise if fish do not move widely among regions and forage locally on prey with distinct FA compositions. In contrast, if fish move widely and forage among regions, they may essentially integrate FAs throughout the systems and spatial patterns will be less apparent; e.g., salmon and trout in Lake Michigan [40].

Fatty acid content and stable isotope ratios of consumers are expected to vary intraspecifically within and across systems in response to different diets, trophic states and allocthonous inputs. In turn, one might expect consistent, strong associations between specific FA indices and stable isotope ratios of individual consumers. However, studies that explored such associations have found mixed results. While there are examples of intraspecific fatty acid–stable isotope associations, within-systems, these may be inconsistent among species due to a) species-specific differences in FA expression and b) limited among-individual variation in diets, and hence limited variation in FA composition and stable isotope ratios [38]. Given that δ^13^C and δ^15^N are very well-established trophic markers, documenting strong and consistent associations with FA can provide further support that diets are driving FA concentrations and justify the use of FAs as trophic markers.

Herein, we describe a study to evaluate stable isotope ratios and relative fatty acid composition of European smelt (*Osmerus eperlanus*) among and within three large lakes. Smelt species (e.g., *O*. *eperlanus* and *O*. *mordax*) are important intermediate trophic level consumers (pelagic planktivores) in large lakes in the Northern Hemisphere. In these systems, it is an open question if a) through broad movement and flexible diets smelt act to integrate lower trophic level production across habitats or b) if they forage more locally and are characterized by region-specific trophic measures. The study lakes and regions within study lakes differ in trophic status, from ultra-oligotrophic to eutrophic. As described above, these productivity differences are expected to be reflected in the stable isotope ratios and fatty acid composition of consumers, such as smelt. We specifically aimed to evaluate three hypotheses. i) Smelts will forage regionally within these lakes which will be reflected in region-specific smelt stable isotope ratios. ii) Stable isotope ratios and fatty acid relative concentrations of smelt will track trophic status of different lakes and regions, with smelts in more productive systems and regions containing lower concentrations of key PUFAs (DHA and EPA), elevated δ^15^N values and lower δ^13^C values. iii) Both types of trophic indicators reflect smelt diet, and they will be correlated at the individual smelt level. In general, we found support for all three hypotheses.

## Methods

### Study systems and species

**Fig 1:**
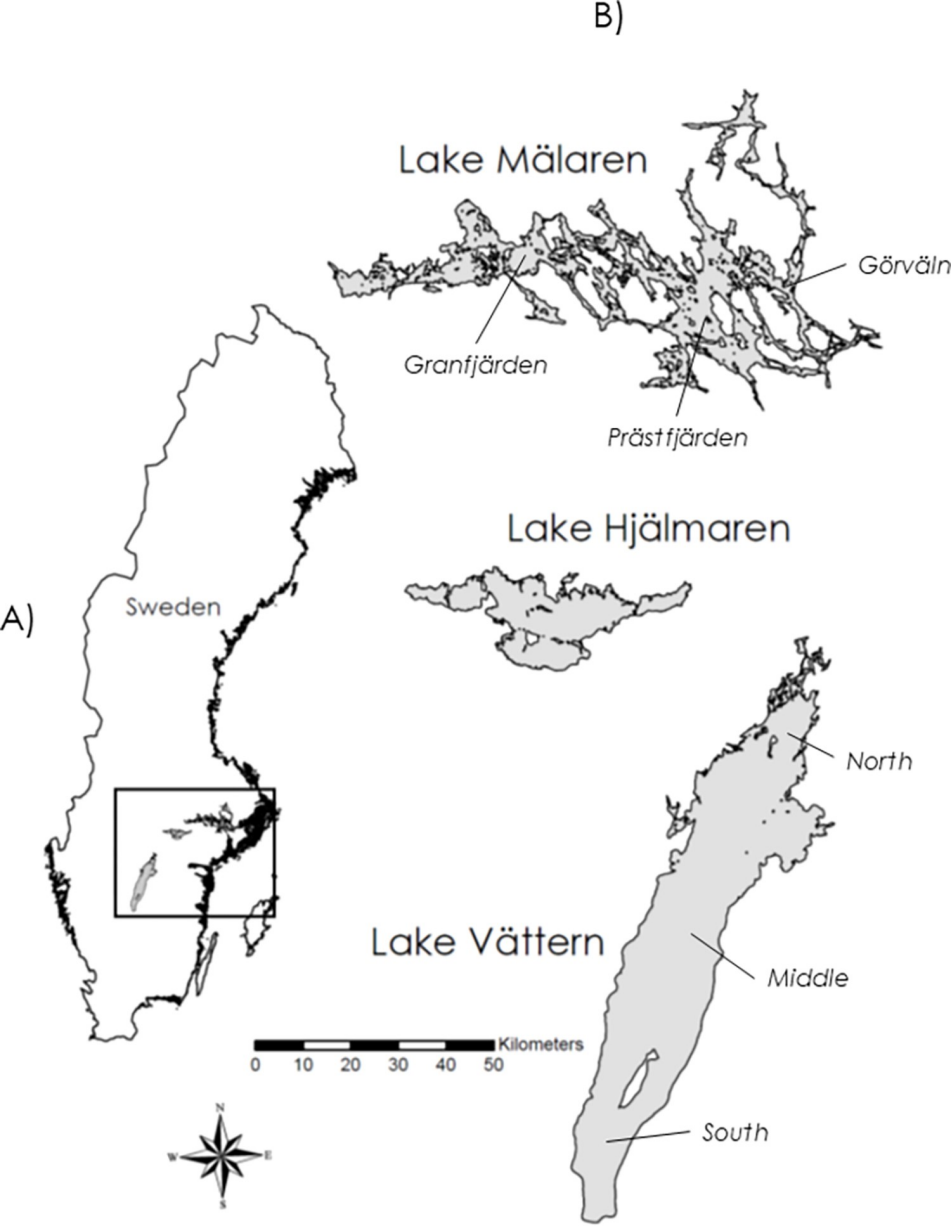
A) Location of study lakes in Sweden. B) Study lakes and regions. Lakes are sized proportionally and scale bar is relevant for all three lakes. The outline of Sweden comes from international.ipums.org (Steven Ruggles, Lara Cleveland, Rodrigo Lovaton, Sula Sarkar, Matthew Sobek, Derek Burk, Dan Ehrlich, Quinn Heimann, Jane Lee. Integrated Public Use Microdata Series, International: Version 7.5 [dataset]. Minneapolis, MN: IPUMS, 2024. https://doi.org/10.1. Minneapolis, MN: IPUMS, 2024. https://doi.org/10.18128/D020.V7.5) and the lake outlines come from a global dataset of large lakes greater than 10 hectares (https://www.arcgis.com/home/item.html?id=0abb136c398942e080f736c8eb09f5c4; Creative Commons Attribution 4.0 International).

The study lakes included three of the four largest lakes in Sweden: Vättern, Mälaren, and Hjälmaren (Fig 1). Vättern is an ultra-oligotrophic lake with very clear water, a relatively small drainage area and a long residence time. It is a long, narrow and fairly open lake. The southern region of the lake is slightly deeper than other regions (max depth, 128m). Moreover, the northern region has a somewhat more complex shoreline, more islands and is adjacent to the lake’s outflow. The majority of the lake (with the exception of the northern archipelago area) is treated as one homogenous basin for both water and fisheries management. Mälaren is the third largest lake in Sweden. It has a very high shoreline complexity, with many islands, bays and distinct basins. Mälaren has a very large drainage area, with two main outflows towards the east into the Baltic Sea with a general productivity gradient from more shallow and productive (eutrophic) areas in the west to deeper and less productive (mesotrophic) areas in the east. The three basins included in this study (Granfjärden, Prästfjärden, and Görväln) are situated from west to east and generally follow this productivity gradient. Hjälmaren is smaller, shallower and more productive (eutrophic) compared to the other two lakes. It has an intermediate drainage area relative to its size and a residence time similar to Mälaren (Table 1). Hjälmaren drains into the western part of Mälaren via a river, Eskilstunaån.

**Table 1:** Characteristics of study lakes and regions. Secchi disc depth from the Swedish National monitoring (means from the period 2010–2020). Agricultural land use in drainage area has been derived from SMHI:s S-HYPE model output per water body (https://www.smhi.se/en/research/research-departments/hydrology/hype-our-hydrological-model-1.7994). Total nitrogen (N) and phosphorous (P) are means from Swedish National Lakes Monitoring Program during April-October 2016. Residence times and drainage area from [44, 65].

Lake	Hjälmaren	Vättern	Mälaren	Mälaren		
Region	entire lake	entire lake	entire lake	Granfjärden	Prästfjärden	Görväln
Max depth (m)	22	126	66	28	56	66
Mean depth (m)	7	40	13	10	17	14
Area (km^2^)	464	1886	1140	76	314	70
Volume (km^3^)	2.6	70.0	-	0.8	5.3	1.0
Drainage area (km^2^)	4,053	6,359	222,603			
Residence time (yr)	4	58	3	-	-	-
Secchi disc depth (m)	1.8	11.0	0.8–5.0	1.2	2.3	2.6
Agriculture (%)	28	14	-	19	21	24
Total N (μg/L)	590	672		617	417	521
Total P (μg/L)	33	4	-	41	20	25

European smelt was chosen as the study species because this species is ecologically important in all three study lakes (and in many other large lakes, estuaries and marine environments in Northern Europe). Smelt populations have historically supported recreational and commercial fisheries in the study lakes and served as the main prey for piscivorous fish such as Arctic char (*Salvelinus alpinus*) in Vättern [41] and pike-perch (*Sander lucioperca*) in Mälaren and Hjälmaren (e.g., [42]). Smelts of small and intermediate sizes (<150 mm) are primarily invertivores, consuming zooplankton and benthic invertebrates. With increasing size, smelts first incorporate fish fry and then include larger fish in their diet, becoming exclusively piscivorous when larger than 225 mm, and are often cannibals [41, 43]. Spatial population structure and movement of smelt in these lakes have not been fully described, and nutritional content of smelts have not been studied within and across systems.

### Field sampling

We collected smelts during late July (Hjälmaren), August (Mälaren), and early September (Vättern) of 2016 as part of an annual assessment program conducted by the Swedish University of Agricultural Sciences. Smelts were collected by overnight sets of multi-mesh gillnets (e.g., [40]) in three basins in Mälaren (Granfjärden, Prästfjärden and Görväln) and one location in the main basin of Hjälmaren, and by nighttime midwater trawling (*R/V* Asterix, 5×12 m trawl opening, 7mm and 5mm cod end mesh; [44, 45]) in three regions of Vättern (south, middle, north). Field collections and handling were conducted with approval of Stockholms Norra Djurförsöksetiska Nämnd (permit N5015) and Sveriges Lantbruksuniversitet Fullmakt fish collection allowance. Upon collection, smelts were either immediately dissected or stored whole and dissected later in the laboratory. Dissected muscle fillets or whole animals were wrapped in aluminum foil, refrigerated during transport and then transferred to a -80°C freezer in the laboratory. Subsequently, samples were shipped to Purdue University on dry ice and then stored at -80°C. Prior to analyses, samples were thawed, a subsample (<0.1 g) of dorsal muscle tissue was obtained for stable isotope analysis and a larger subsample (0.5–3.0 g) of muscle tissue was obtained for fatty acid quantification.

### Stable isotope analysis

For stable isotope analyses, we dried the samples in an oven (60°C) and homogenized using a mortar and pestle. We then shipped samples to the University of California-Davis Stable Isotope Facility (stableisotopefacility.ucdavis.edu/), where C and N isotope content of 1.00–1.28 mg dry material was analyzed using an elemental analyzer (PDZ Europa ANCA-GSL) interfaced to a continuous flow isotope ratio mass spectrometer (PDZ Europa 20–20). Isotope ratios were expressed using delta notation relative to international standards: Vienna Pee Dee beleminite (δ^13^C) and atmospheric N_2_ (δ^15^N). Samples were analyzed with four reference samples, allowing for calculation of measurement variation (SD): bovine liver (δ^13^C = 0.15‰; δ^15^N = 0.04‰), glutamic acid (δ^13^C = 0.09‰; δ^15^N = 0.08‰), enriched alanine (δ^13^C = 0.03‰; δ^15^N = 0.03‰), and nylon 6 (δ^13^C = 0.04‰; δ^15^N = 0.03‰).

### Fatty acid analysis

We analyzed fatty acids at Purdue University’s Department of Forestry and Natural Resources following described methods [46]. In brief, we extracted lipids using 2:1 chloroform:methanol + 0.01% butylated hydroxytoluene solution and centrifugation [47], and prepared FA methyl esters through drying with N gas, resuspension in dichloromethane, a transesterification reagent (170 ml methanol, 2.55 ml concentrated sulfuric acid) and re-extraction with hexane [for more details, see: 48]. Then, we measured FA composition using a Shimadzu gas chromatograph model GC-2010 with flame ionization detector, autoinjector, and EZStart software version 7.4 (Shimadzu Corp.). We analysed samples together with standards: Sigma-Aldrich 47563-U Supelco, Sigma-Aldrich 72332-5G-F, Sigma-Aldrich 74723, Sigma-Aldrich 68824, Nu-Chek-Prep U-48-M (Nu-Chek-Prep, Inc.), Nu-Chek-Prep U-83-M, Nu-Chek-Prep A-633, Nu-Chek-Prep A-637, Nu-Check-Prep U-101-M, Nu-Chek-Prep U-102-M, Matreya 1114 (Matreya, LLC.), and we included menhaden fish oil as an internal reference sample. To limit the influence of any variation in measurement effectivity, we simultaneously analysed samples from multiple locations during each day of analysis. We followed previous recommendations [26] and expressed each fatty acid’s composition as percentage composition of total FA mass. For subsequent analysis, we focused on those FAs that represented >1% of total FA mass by sample median.

### Data analysis

To limit the influence of individual size on FA and isotopic variation, we analyzed smelts between 75 mm and less than 125 mm total length (approximate ages 1–2; [43, 49]). The vast majority of smelt collected were within this range and some smaller individuals collected in Vättern were not included in statistical analyses (see below). To visualize how smelts varied isotopically by region, we plotted individual smelt δ^13^C values versus δ^15^N values. Similarly, we used a principal components analysis (PCA) to visualize how smelt from different regions differed in FA profiles. Prior to conducting the PCA, we took the arcsine square root of relative percentage composition of each FA to more closely approximate parametric distributions, and we included the twelve FAs with median percentage composition of at least 1% (see S2 Table). We alsoe calculated ω3:ω6 ratio for each smelt based upon these abundant FAs. These analyses and subsequent statistical analyses were conducted using SPSS v26.0 [50].

We developed a series of general linear models (GLMs) to consider how smelt δ^13^C, δ^15^N, ω3:ω6, %DHA and %EPA varied by region (all percentages were arcsine-square root transformed prior to analyses). For each of these GLMs, we included region (Hjälmaren, Mälaren-Granfjärden, Mälaren-Prästfjärden, Mälaren-Görväln, Vättern-North, Vättern-Middle, or Vättern-South) as a fixed factor and individual length as a covariate, along with an interaction term (region × length). We conducted post hoc comparisons using Šidák multi-comparisons to evaluate which regions differed from each other. We developed these models and post hoc analyses to compare regional effects rather than directly assessing potential influences of various environmental factors (e.g., Secchi depth; see Table 1) because with only seven regions we would be unable to effectively evaluate such separate environmental effects.

While our focus was to evaluate across-lake and -region differences in FA composition and stable isotope ratios, we also explored how the two most abundant PUFAs, DHA and EPA, and a cumulative PUFA index, ω3:ω6, varied among individual smelt within each region. The use of these two PUFAs and ω3:ω6 as trophic indices is not as well established as δ^13^C and δ^15^N. Moreover, the range of fatty acid concentrations and stable isotope ratios observed across regions and lakes far exceeded variation within a region. Thus, to elucidate associations (and the consistency of such associations) among individual smelt relative concentrations of dominant PUFAs and stable isotope ratios, we evaluated regions independently. To this end, we used region-specific Spearman rank correlations to associate individual smelt DHA, EPA and ω3:ω6 with a) each other and b) δ^13^C and δ^15^N (to explore associations with well-established trophic indices). For each correlation, we evaluated significance (α = 0.05) before and after conducting a Šidák correction for multi-comparisons (adjusted for number of regions, m = 7).

## Results

**Fig 2:**
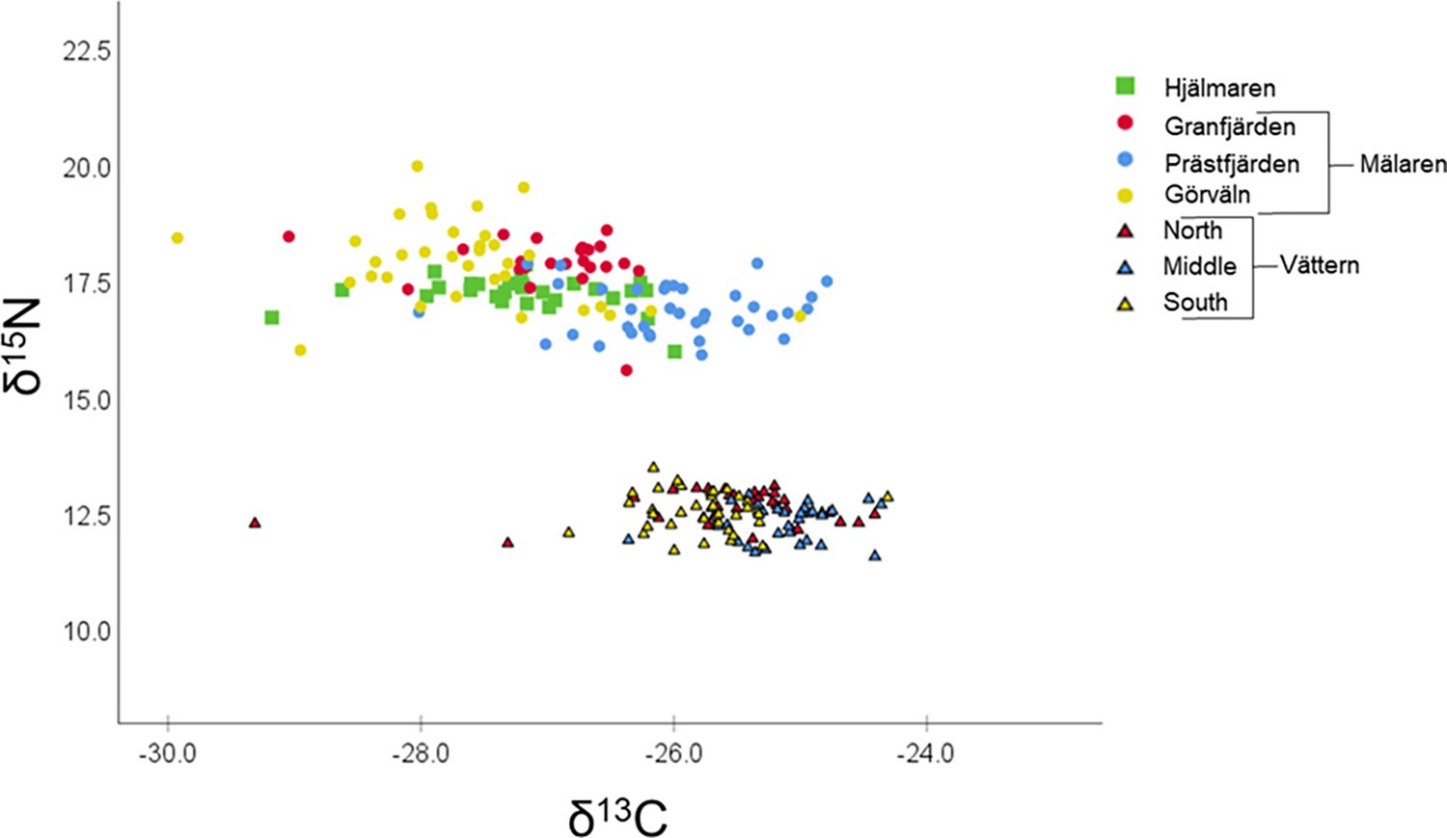
Individual European smelt δ^13^C versus δ^15^N. Plot includes smelts with total lengths between 75–125 mm. Shape and color of points correspond to smelt captured in different lakes and regions, respectively.

**Fig 3:**
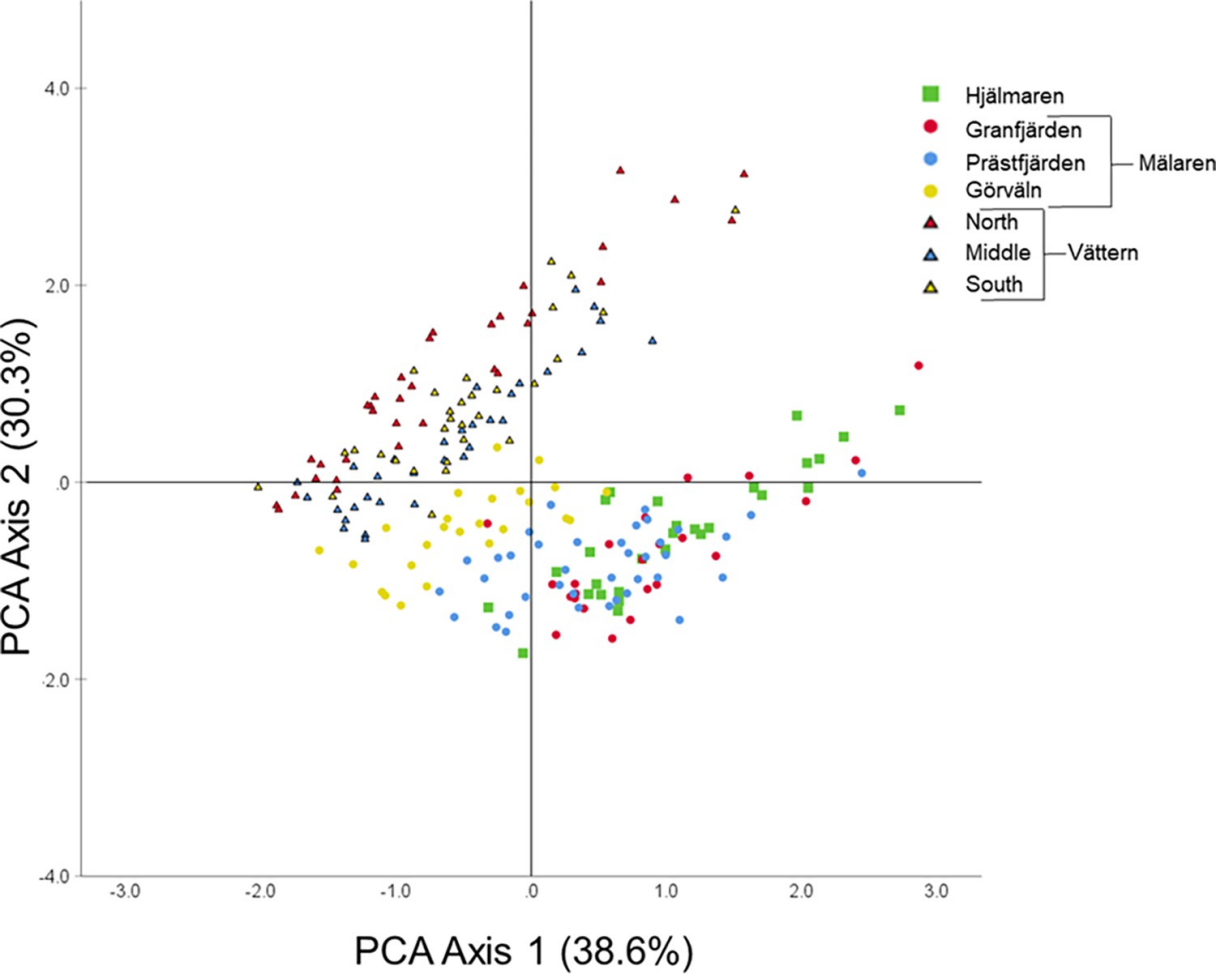
Individual European smelt (75–125 mm) fatty acid principal component analysis (PCA) axis 1 (38.6% of variation) versus axis 2 (30.3% of variation). Shape and color of points correspond to smelt captured in different lakes and regions, respectively.

**Fig 4:**
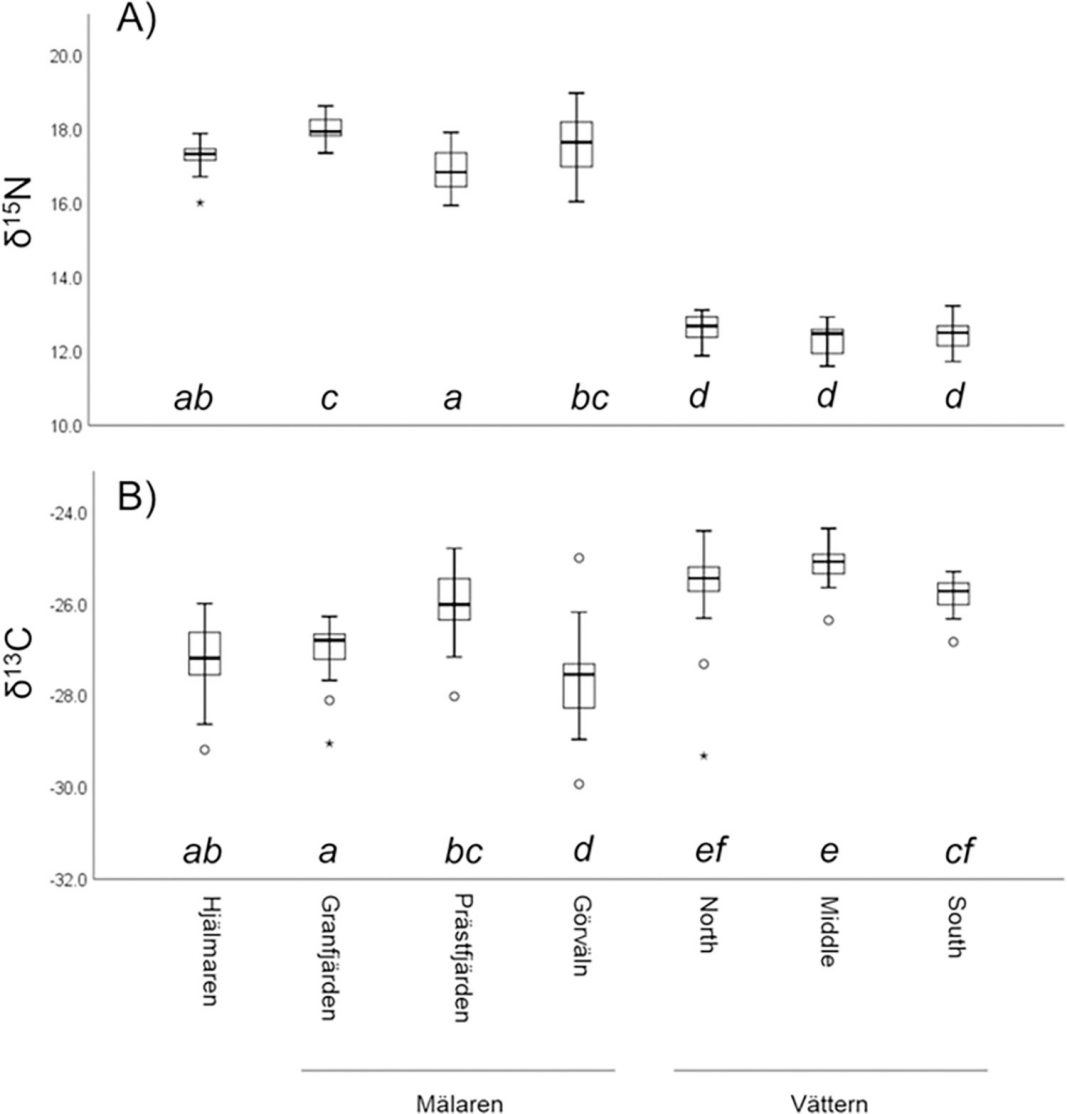
Box plots of European smelt (A) δ^15^N (‰) and (B) δ^13^C (‰) from different regions. Box plot distributions are for all smelt between 75–125 mm and are unadjusted for length. However, shared letters below box plots indicate that regions do not differ significantly based upon Šidák corrected multi-comparisons, following GLMs which included length as a covariate.

Based on both stable isotope ratios (Fig 2) and FA profiles (Fig 3), smelts between 75–125 mm total length differed among the three study lakes and among regions in Mälaren. In general, smelts from the most oligotrophic lake, Vättern, displayed lower δ^15^N values (i.e., ^15^N depletion) and greater δ^13^C values (i.e., ^13^C enrichment), relative to the more productive Hjälmaren and Mälaren (Figs 2 and 4). Based on the GLMs, smelt δ^13^C and δ^15^N varied by region of capture and individual length. Region, total length and the interaction between region and length were all statistically significant (p < 0.05): δ^13^C, adjusted R^2^ = 0.66, F_6,188_(region) = 4.04 p<0.0005, F_1,188_(length) = 25.15 p<0.0005, F_6,188_(interaction) = 3.29 p = 0.004; δ^15^N, adjusted R^2^ = 0.97, F_6,188_(region) = 19.33 p<0.0005, F_1,188_(length) = 13.80 p<0.0005, F_6,188_(interaction) = 2.17 p = 0.048 (see S1 Table). Based on post hoc comparisons using Šidák multi-comparisons (evaluated at a mean length of 97.31 mm), δ^13^C and δ^15^N values of smelt from the three regions of Vättern were generally similar to each other, but distinct from smelt from different regions in Mälaren and Hjälmaren. In contrast, δ^13^C and δ^15^N of smelt from Mälaren generally differed across the three study regions (Fig 4).

We detected 28 different FAs in smelt tissue, and of these twelve FAs contributed with at least 1% of total fatty acid mass (by median) and were included in the subsequent analyses. The most abundant fatty acids by percentage were DHA (21.1%), C16:0 (20.2%) and EPA (14.7%; S2 Table). Principal components analysis (PCA) indicated clear differentiation among some regions, with North–Vättern, Middle–Vättern, and South–Vättern grouping together, Hjälmaren, Mälaren-Granfjärden and Mälaren-Prästfjärden grouping together and Mälaren-Görväln exhibiting FA compositions intermediate of these two broader groupings (Fig 3). Most of the variation described by the PCA was incorporated in the first two components (38.6% and 30.3% of variation, respectively). In particular, DHA loaded strongly and negatively on the first component (-0.957) and had very limited influence on the second component (-0.077; See S2 Table).

**Fig 5:**
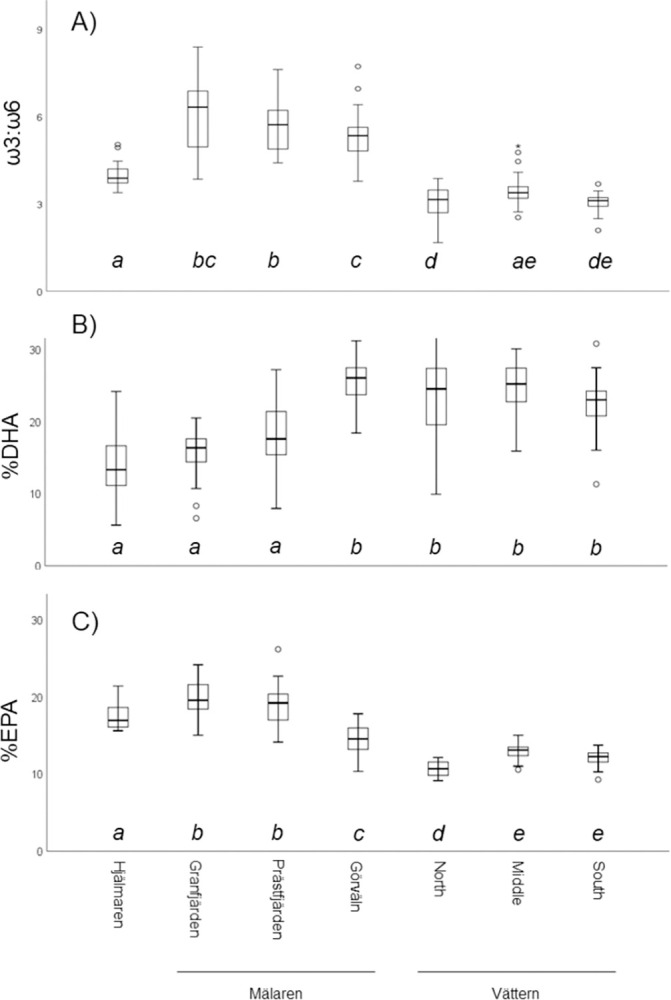
Box plots of European smelt ω3:ω6 fatty acid ratios (A), percent EPA (B) and percent DHA (C) from different regions. Box plot distributions are unadjusted for length. However, shared letters below box plots indicate that regions do not differ significantly based upon Šidák corrected multi-comparisons, following GLMs which included length as a covariate.

General linear models to consider how smelt ω3:ω6, %DHA and %EPA varied by region of capture and individual total length accounted for a great deal of variation in these FA indices and indicated significant effects of region, length and the interaction between region and length: ω3:ω6 (response variable), adjusted R^2^ = 0.75, F_6,196_(region) = 6.03 p<0.001, F_1,196_(length) = 0.784 p = 0.377, F_6,196_(interaction) = 3.62 p = 0.002; %DHA, adjusted R^2^ = 0.56, F_6,196_(region) = 2.96 p = 0.009, F_1,196_(length) = 39.22 p<0.0005, F_6,196_(interaction) = 2.15 p = 0.050; %EPA, adjusted R^2^ = 0.84, F_6,196_(region) = 5.73 p<0.0005, F_1,196_(length) = 5.49 p = 0.20, F_6,196_(interaction) = 6.84 p<0.0005 (see S1 Table). Based on post hoc comparisons using Šidák multi-comparisons, smelts in different regions of Vättern generally displayed low and similar ω3:ω6 values. In contrast, smelts from Mälaren displayed relatively high ω3:ω6 values, and smelts from Hjälmaren displayed intermediate ω3:ω6 values. Smelts from the three regions of Vättern and Mälaren-Görväln generally exhibited similar %DHA and %EPA values. Similarly, smelts from Hjälmaren, Mälaren-Granfjärden and Mälaren-Prästfjärden generally exhibited similar %DHA and %EPA values (Fig 5).

**Fig 6:**
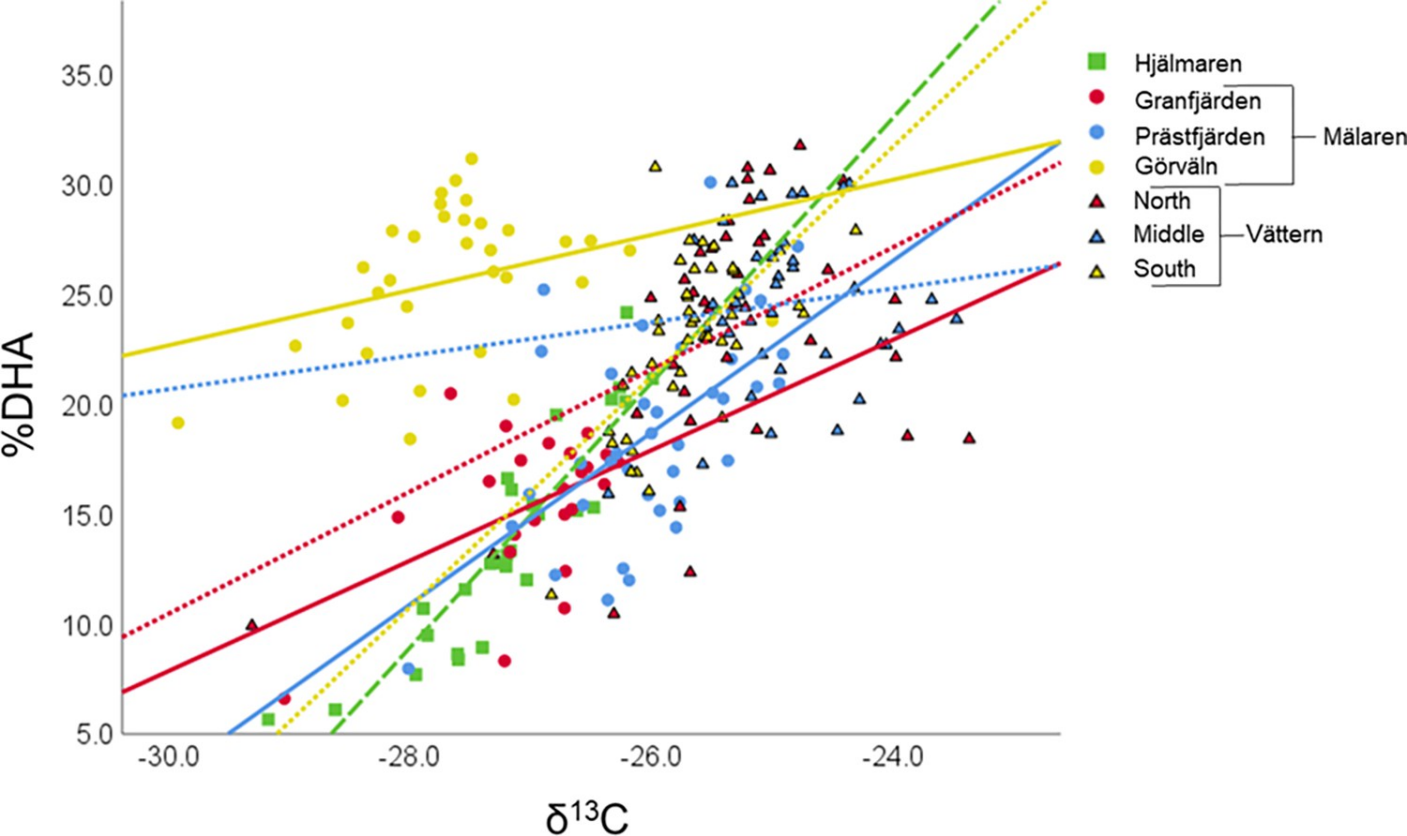
Scatter plots of region-specific individual smelt (75–125 mm) percent DHA versus δ^13^C values (‰). Shape and color of points correspond to smelt captured in different lakes and regions, respectively. Significant associations were identified via Spearman rank correlations (see Table 2). Linear relationships depicted with region-specific trendlines for purpose of visualization (Hjälmaren, long dashed line; Mälaren, solid lines; Vättern, short dashed lines).

Region-specific Spearman rank correlations to associate %DHA, %EPA and ω3:ω6 of individual smelt with δ^13^C and δ^15^N, suggested that several associations were consistent across regions (Table 2 and Fig 6). In particular, %DHA and %EPA were generally negatively associated with each other. In addition, %DHA was consistently positively associated with δ^13^C. Within specific regions, %EPA was significantly associated with other metrics (e.g., negative associations with δ^13^C in two regions and negative associations with δ^15^N in two other regions). However, in contrast to %DHA such associations with EPA were generally not coherent across regions. Within regions, ω3:ω6 tended to be positively associated with δ^13^C and %DHA, but when combining all individuals across regions, these associations were either negative (δ^13^C and ω3:ω6) or absent (%DHA and ω3:ω6).

**Table 2:** All lakes and region-specific Spearman rank correlation coefficients (ρ) for individual smelt percent concentrations of A) DHA and B) EPA correlated with individual smelt δ^13^C (‰), δ^15^N (‰), percent EPA, and percent DHA. Note that all lake and region specific-samples sizes (n) are ranges because some individuals were not successfully analyzed for stable isotopes. Correlation coefficients in italics are significant at unadjusted α = 0.05 level, while correlation coefficients in bold-underlined are significant after Šidák corrections (m = 8).

A) Spearman rank correlations (ρ) with %DHA						
		δ^13^C	δ^15^N		%EPA	ω3:ω6
All lakes (n = 202–210)		**+0.510**	**-0.300**		**-0.620**	-0.100
Hjälmaren (n = 26–28)		**+0.931**	-0.123		+0.047	**+0.600**
Mälaren	Granfjärden (n = 22–23)	+0.263	+0.269		+0.055	+0.335
	Prästfjärden (n = 36–38)	**+0.515**	*+0*.*354*		**-0.744**	+0.149
	Görväln (n = 25)	+0.392	+0.126		**-0.738**	+0.375
Vättern	North (n = 32–34)	**+0.683**	+0.326		**-0.451**	**+0.975**
	Middle (n = 31–32)	*+0*.*383*	+0.186		**-0.580**	*+0*.*383*
	South (n = 30)	**+0.532**	+0.228		**-0.564**	-0.097
B) Spearman rank correlations (ρ) with %EPA						
		δ^13^C	δ^15^N		%DHA	ω3:ω6
All lakes (n = 202–210)		**-0.505**	**+0.630**		**-0.620**	**+0.676**
Hjälmaren (n = 26–28)		+0.130	-0.111		+0.047	+0.232
Mälaren	Granfjärden (n = 23–24)	+0.260	+0.137		+0.055	+0.074
	Prästfjärden (n = 36–38)	**-0.471**	-0.260		**-0.744**	-0.097
	Görväln (n = 25)	-0.292	-0.279		**-0.738**	**-0.672**
Vättern	North (n = 32–34)	-0.250	**-0.481**		**-0.451**	*-0*.*355*
	Middle (n = 31–32)	-0.329	*-0*.*392*		**-0.580**	-0.234
	South (n = 32)	*-0*.*463*	-0.063		**-0.564**	+0.149
C) Spearman rank correlations (ρ) with ω3:ω6						
		δ^13^C	δ^15^N		%DHA	%EPA
All lakes (n = 202–210)		**-0.390**	**+0.717**		-0.100	**+0.676**
Hjälmaren (n = 26–28)		**+0.517**		-0.007	**+0.600**	+0.232
Mälaren	Granfjärden (n = 23–24)	+0.138		-0.327	+0.335	+0.074
	Prästfjärden (n = 36–38)	-0.046		+0.030	+0.149	-0.097
	Görväln (n = 25)	+0.289		+0.232	+0.375	**-0.672**
Vättern	North (n = 32–34)	**+0.643**		+0.322	**+0.975**	*-0*.*355*
	Middle (n = 31–32)	+0.065		-0.273	*+0*.*383*	-0.234
	South (n = 32)	**+0.559**		+0.135	-0.097	+0.149

## Discussion

In general, observed spatial patterns of European smelt fatty acid relative concentrations and stable isotope ratios were consistent with our hypotheses. Relative to our first hypothesis, regional differences in stable isotope ratios of smelts in Mälaren indicate that individuals in this lake do not continuously migrate extensively between the different basins. Rather, these differences suggest that smelt feed regionally, at least during the weeks to months before capture, and thereby develop a region-specific isotope signature. Mälaren is a spatially complex lake with many embayments, islands and basins of varying depth. This complexity likely leads to seasonal restrictions in the movement of fish, especially for a physiologically cold-water adapted species like smelt. In contrast to smelt in different regions of Mälaren, smelt in different regions of Vättern expressed fairly similar FA patterns and stable isotope values. Vättern is less spatially complex than Mälaren, potentially facilitating broad movement throughout the system. Alternatively, trophic differences among regions of Vättern may simply not be sufficiently distinct to allow for detection of regional foraging based upon stable isotope and fatty acids.

Consistent with our second hypothesis, FA and stable isotope values of smelt were seemingly associated with system productivity. Smelts from ultra-oligotrophic Vättern were characterized by relatively depleted ^15^N, enriched ^13^C, and high %PUFA and %DHA. In contrast, smelt from eutrophic Hjälmaren were characterized by high δ^15^N values, low δ^13^C values and low relative %PUFA and %DHA concentrations. In Mälaren, a gradient was generally evident from more eutrophic Granfjärden in the west to the more nutrient-poor Görväln in the east. Specifically, in Mälaren %PUFA and %DHA of smelt increased from west to east. Stable isotope ratios of smelt in Mälaren also varied among regions, but these did not align in a manner entirely consistent with expectations based on system productivity. Instead, these patterns may be reflective of regional differences in water residence times and allochthonous sources.

In regards to our third hypothesis, within regions there were consistent associations between FA and stable isotope measures of individual smelt. Specifically, at the individual smelt level %DHA (the most abundant fatty acid) had strong positive influence on overall %PUFA, but %DHA and %EPA were generally negatively associated. In addition, within all regions individual smelt δ^13^C was positively associated with DHA, likely reflecting diet differences over the relatively narrow length range considered and pointing to the potential utility of DHA concentration as a trophic marker for smelt.

### Stable isotope patterns

Stable isotope ratios of consumers (δ^13^C, δ^15^N) are reflective of a variety of underlying processes, including diets, dominant primary producers, and isotopic sources supporting the base of the food web. Past diet studies demonstrate that within the size range we considered, smelts in these lakes are primarily zooplanktivorous [43]. However, they are also known to consume pelagic macroinvertebrates (e.g., *Mysis*) and benthic invertebrates (e.g., Amphipoda, Diptera), with increased consumption of such larger prey as smelts increase in size and age. In short, while prey consumption patterns were likely broadly similar among the three study lakes, we cannot exclude the contribution of differential prey consumption patterns contributing to differences in stable isotope ratios. In addition, system productivity may be related to the number of trophic levels supporting consumer production, with lower number of trophic levels supporting top predators in less productive systems as compared to more productive systems [51]. That is, there may be additional trophic steps to transfer energy from primary producers to higher level consumers in more productive systems. Relatively low δ^15^N of smelt in Vättern may partially be related to this association between system productivity and consumer trophic level (i.e., low number of trophic steps in this ultraoligotrophic lake).

While differential prey consumption patterns and food web structure may partially contribute to stable isotope ratio differences among lakes and regions, smelt stable isotope ratios among lakes and regions aligned with differences in allochthonous inputs and relative system productivity. In systems dominated by cyanobacteria (e.g., some highly eutrophic systems), incorporation of atmospheric N would be expected to contribute to ^15^N-depleted particulate organic matter [52, 53]. However, across systems of differing productivity the opposite pattern is overwhelmingly evident. That is, several past studies indicate that ^15^N is relatively enriched in biota in more productive systems (e.g., [8, 54]). Similarly, biotic δ^15^N tends to be higher in coastal lake and marine regions with high riverine influences (e.g., [16, 55]) and is particularly high in regions receiving drainage from highly anthropogenically influenced watersheds [15]. While the association between system productivity and δ^13^C of biota is less consistent, in general ^13^C tends to be relatively depleted in more productive systems [19] and in habitats with more riverine influence [7, 16, 19, 20]. Eutrophication-induced decreases in water clarity can contribute to reduced relative benthic production and more reliance of pelagic pathways by consumers [22], which is expected to lead to lower δ^13^C consumer values. However, some studies have found the opposite association between eutrophication and consumer δ^13^C (e.g., [9, 21]). Nonetheless, smelt stable isotope ratios across study lakes and regions adhered to general δ^13^C and δ^15^N expectations related to the influence of system productivity. Smelt from all three regions of ultraoligotrophic Vättern were characterized by relatively low δ^15^N values and high δ^13^C values, while smelts from eutrophic Hjälmaren were characterized by relatively high δ^15^N values and low δ^13^C values. In addition, within Mälaren smelt isotopic values generally aligned with this expectation along a gradient from the more productive west region to the less productive east region. While Hjälmaren and western Mälaren are connected by a regulated stream and reservoir system, it is not expected that many smelts move between the two systems. Instead, the similarity in isotope values between smelts from Hjälmaren and western Mälaren is likely related to similar allocthonous inputs and eutrophication status. Of note, smelts from Görväln were characterized by lower δ^13^C and slightly higher δ^15^N than expected based on productivity patterns. The reasons for this discrepancy are not entirely clear, but Görväln is a hydrologically complicated basin with drainage contributions from diverse watersheds, also receiving significant part of its water from a more eutrophic, high alkalinity basin to its north [56].

Differential water residence times likely also contributed to patterns of smelt N stable isotope ratios. In general, longer residence times are expected to allow for greater relative nitrogen retention [57] and longer retention, along with relatively small catchments areas, are expected to contribute to relatively high incorporation of N from over-lake deposition into aquatic food webs. This would be expected to contribute to lower δ^15^N for consumers in systems with greater residence times and low catchment area to lake volume ratios. To this point, Vättern has a very long residence time and low catchment area ratio relative to its volume, as compared to Hjälmaren and Mälaren. Consistent with this, smelt in Vättern had consistently lower δ^15^N values. Further, application of a hydrodynamic model of Mälaren is indicative of differential residence times among the various basins of this lake [56]; with the shortest residence time for water in the western region (i.e., Granfjärden), particularly high residence time for Prästfjärden and intermediate for Görväln. The lower δ^15^N of smelt in Prästfjärden (relative to Granfjärden and Görväln) may be reflective of these differences in water residence times. Future studies could more fully explore stable isotope ratio variation among multiple trophic levels of study lakes. In particular, in spatially complex Mälaren regional isotope measures could better elucidate local food webs and the spatial structure of the system-wide food web.

Fatty acid patterns and implications.

Consumer fatty acid patterns reflect a combination of diet, environmental factors (e.g., temperature), and physiological processes. Some FAs can be modified by consumers and different fatty acids may be favorably sequestered, utilized and retained by consumers. Thus, entire FA profiles do not simply reflect diets. In fact, consumer species differ in their prioritized incorporation and utilization of various FAs, and within systems there are generally clear species-specific fatty acid profiles among consumers [26, 58]. To this point, the relative concentrations of different FAs reported for smelt in this study are similar to what other studies have documented for European and rainbow smelt, with smelt consistently characterized by particularly high concentrations of DHA (e.g., [23, 26, 58]). In addition, various freshwater fish species, including smelt, have been demonstrated to accumulate specific PUFAs and in particular accumulate DHA; such that within a system fish DHA concentrations are greater than DHA concentrations of both primary producers and the prey directly consumed by fish [26, 59]. Nonetheless, consumers have limited ability to modify PUFAs and intra-specific differences in concentrations of PUFAs are expected to largely reflect differences in FAs obtained through diet.

Across lakes and regions, relative concentrations of total PUFAs and DHA in smelts were higher in systems characterized by relatively low productivity, while relative concentrations of EPA were higher in systems characterized by relatively high productivity. Primary producers vary in their relative concentrations of different PUFAs, with taxa such as diatoms expressing relatively high concentrations of EPA, dinoflagellates expressing high DHA concentrations, and chlorophytes and cyanobacteria synthesizing neither EPA or DHA [30, 33]. Thus, differences in primary producer communities among lakes and regions are likely reflected in smelt fatty acid profiles. In addition, within systems invertebrates often differ in fatty acid composition. For example, past studies [59, 60] found that littoral macroinvertebrates and pelagic cladocerans had particularly low relative concentrations of DHA, while pelagic copepods expressed high relative concentrations of DHA. Similarly, across several Finnish lakes [60], found particularly high concentrations of DHA in copepods (especially *Heterocope* and *Limnocalanus*), and *Mysis*, and low concentrations in cladocerans. While [59] also found slightly lower concentrations of EPA in littoral macroinvertebrates as compared to zooplankton, these differences were not very pronounced. Moreover [59], found very similar EPA concentrations in cladocerans and copepods, and [60] did not observe differences in relative EPA concentrations among cladocerans, copepods and littoral invertebrates. Thus, consumption of high levels of littoral benthic invertebrates and cladocerans may be expected to contribute to relatively low concentrations of DHA in invertivorous fish tissue, while greater reliance on pelagic planktonic invertebrate prey and in particular consumption of copepods and *Mysis* may contribute to relatively high concentrations of DHA in fish. In general, more oligotrophic systems may be expected to be dominated by DHA-rich copepods and *Mysis*, and consistent with our observations, consumers in more oligotrophic systems would be expected to contain greater DHA concentrations.

Stomach content analyses of smelts across our three study lakes reveal both among- and within-system diet variation. A past study [41] presented a thorough stomach content analysis of 382 smelt collected in Vättern, May-December 1973–2009. They showed that smelts <100 mm in length relied on a diverse diet with *Bosmina* being the most common prey volumetrically and in terms of frequency. Smelts between 100–150 mm switched to larger prey, with *Mysis* constituting 61% of diet by volume, and once smelt reached 150 mm they switched to primarily consume fish as prey. Another study [43] examined smelt stomach contents from Hjälmaren, Mälaren (collections near Prästfjärden and Görväln), and Vättern (collections in northern and middle Vättern) for the period September-November 1989–1990. In all three lakes, they found that smelts consumed a diverse diet including cladocerans, copepods, Diptera, Amphipoda and *Mysis*. Interestingly, they found that on a relative biomass basis age-1 and -2 smelts in Vättern consumed a greater proportion of expected DHA-poor prey (cladocerans, Diptera) than in Hjälmaren and Mälaren. In addition, across all three lakes they showed that as smelt increased in size and age they consumed a greater proportion of relatively large prey, including *Mysis*.

We found clear differences in fatty acid concentrations of individual smelts within the same lake and region (see broad ranges in Fig 5). DHA was the most abundant PUFA and the relative concentration of DHA was associated with overall %PUFA; as relative DHA concentration increased, overall %PUFA increased. In contrast, %EPA was not consistently associated with overall %PUFA, and %EPA and %DHA were either not associated or negatively associated. Specifically, within the more oligotrophic regions (all regions except Hjälmaren and Granfjärden) there was a negative association between %DHA and %EPA. The mechanisms underlying such associations are not entirely clear, but may reflect a combination of differences in dominant primary producers among regions, diet variation and differences in FA conversion and retention by smelts among regions.

The ω3:ω6 index has been used to assess benthic versus pelagic reliance, as well as relative terrestrial influence [36–38, 61, 62]. This index is generally expected to be relatively low in terrestrial sources, due in part to relatively high ω6 FA concentrations in such sources [39]; but see caution by [30]. Several past studies have indicated that ω3:ω6 tends to be lower in littoral producers and consumers than pelagic producers [36–38, 61]. In this study, the index was relatively high for smelts from Mälaren, low for smelts from Vättern and intermediate for smelts from Hjälmaren. It does not seem plausible that high terrestrial inputs to Vättern would be a primary mechanism leading to differences in smelt ω3:ω6 among lakes. Instead, we suggest that these differences likely are suggestive of differential reliance on benthic/littoral versus pelagic pathways among lakes. However, the exact mechanisms leading to this pattern are unclear. Potentially, seasonal bottom water hypoxia in some basins of Mälaren, may limit access to benthic prey. Further, high water clarity in Vättern may contribute to high benthic primary production and increased importance of benthic prey in this oligotrophic system.

Differences among lakes and regions led ω3:ω6 of individual smelts across all regions to be negatively associated with δ^13^C and positively associated with δ^15^N and %EPA. However, within individual regions these associations were quite different, including several positive ω3:ω6 - δ^13^C associations. Such within-region associations suggest that smelts that rely more on benthic resources in a given region (i.e., high δ^13^C) also express high ω3:ω6. The inconsistency of ω3:ω6 - δ^13^C associations when considering across regions versus within regions points to the complexity of using this index in assessing production pathways [30].

Both across and within lakes and regions, there were consistent positive associations between individual smelt %DHA and δ^13^C. Within regions, both of these measures were also positively associated with individual smelt length. While we only considered a relatively narrow range of lengths (50 mm range), within this range smelt clearly express differential prey consumption patterns; with increased reliance on macroinvertebrates, including *Mysis*, with increasing smelt size [41, 43]. If larger smelt switched to consume more littoral benthic macroinvertebrates, this would likely be reflected in increased δ^13^C values; as greater reliance on benthic prey and production pathways is expected to be reflected in increased δ^13^C values. However, increased reliance on such benthic prey would likely contribute to consumption of relatively DHA-poor prey, which is not consistent with the positive association between percent DHA and δ^13^C. Plausibly, older smelt may partially accumulate and retain DHA obtained from diets consumed earlier in life when smaller. More likely, the positive DHA—δ^13^C association reflects increased consumption of *Mysis* as smelt grow. As omnivores which consume both pelagic and benthic prey, *Mysis* often express higher δ^13^C values than pelagic zooplankton (e.g., [63]), and *Mysis* are characterized by relatively high DHA concentrations [26].

Within- and across-lake variation in smelt PUFA content likely has implications for consumers of smelt, from piscivorous fish to humans. In many lakes, smelt are key prey for piscivorous fish, including pikeperch in Hjälmaren and Mälaren [42] and Arctic char in Vättern [41]. In particular, in Vättern Arctic char less than 400 mm consume relatively high biomass of smelt, whereas larger Arctic char consume more vendace (*Coregonus albula*; [41]). Similarly [42], reported that in Mälaren pikeperch less than 485.5 mm rely on smelt as the dominant prey while larger pikeperch in part continue to consume smelt but also switch to rely more on vendace. In Mälaren, outside of spawning migrations pikeperch display limited movement among basins [64]. Given the observed variation in fatty acid content among smelt in various regions of Mälaren, it follows that pikeperch remaining and feeding on smelt in distinct regions may also display regional FA variation within this lake, and this could be evaluated in future studies.

In conclusion, spatial patterns of European smelt stable isotope ratios and fatty acid concentrations were largely consistent with initial hypotheses. Within-lake stable isotope patterns in a complex lake (Mälaren) were highly suggestive that smelts did not migrate broadly and did not feed integratively throughout this lake during the period prior to field sampling. It is unclear if the lack of regional differences in smelt stable isotope ratios in more open Vättern are reflective of more broad movement and foraging in this lake, or if limited spatial variation of lower trophic level stable isotope ratios simply precluded detection of regional differences in this lake. Overall, smelt stable isotope ratios and fatty acid relative concentrations among lakes and regions appeared to track differences in allochthonous inputs and productivity differences, with smelt in more productive systems and regions generally characterized by low %DHA and δ^13^C values, along with elevated δ^15^N values. While fatty acid concentrations are not expected to directly track fatty acid composition of diet, consistent strong relationships between individual smelt %DHA and δ^13^C are highly indicative of diet affecting fatty acid relative concentrations and point to the utility of %DHA as a trophic indicator for smelt.

## Supporting information

S1 Fig Individual European smelt δ^13^C values (‰) versus δ^15^N values (‰). Plot includes all sizes of smelt captured during study, but does not include two outliers. Color and shape of points correspond to smelt captured in different regions and lakes, respectively. Note that all smelt with δ^15^N values less than 10.1 were less than 75 mm in length and removed before subsequent statistical analysis.
(TIF)

S1 Table General Linear Model results evaluating smelt A) stable isotope ratios (δ^13^C, δ^15^N; all values ‰), B) ω3:ω6 fatty acid ratios, and C) fatty acids (arcsin sqrt transformed %DHA, %EPA). Region-specific model means (±SE) were evaluated at smelt total length of 97.31 mm for stable isotopes and 97.13 mm for fatty acids. Effect coefficients (±SE) are provided for intercept, length (mm), individual regions (relative to Vättern-South) and the interaction between length and region (×Length).
(DOCX)

S2 Table Principal component analysis (PCA) loadings and mean relative concentrations of 12 fatty acid concentrations measured in European smelt. (DOCX)
