# Supplementary figures and images for: Stable isotope and fatty acid variation of a planktivorous fish among and within large lakes
Source: PLoS One. 2024 Jul 22;19(7):e0304089. doi: 10.1371/journal.pone.0304089 (PMC11262694; doi:10.1371/journal.pone.0304089)

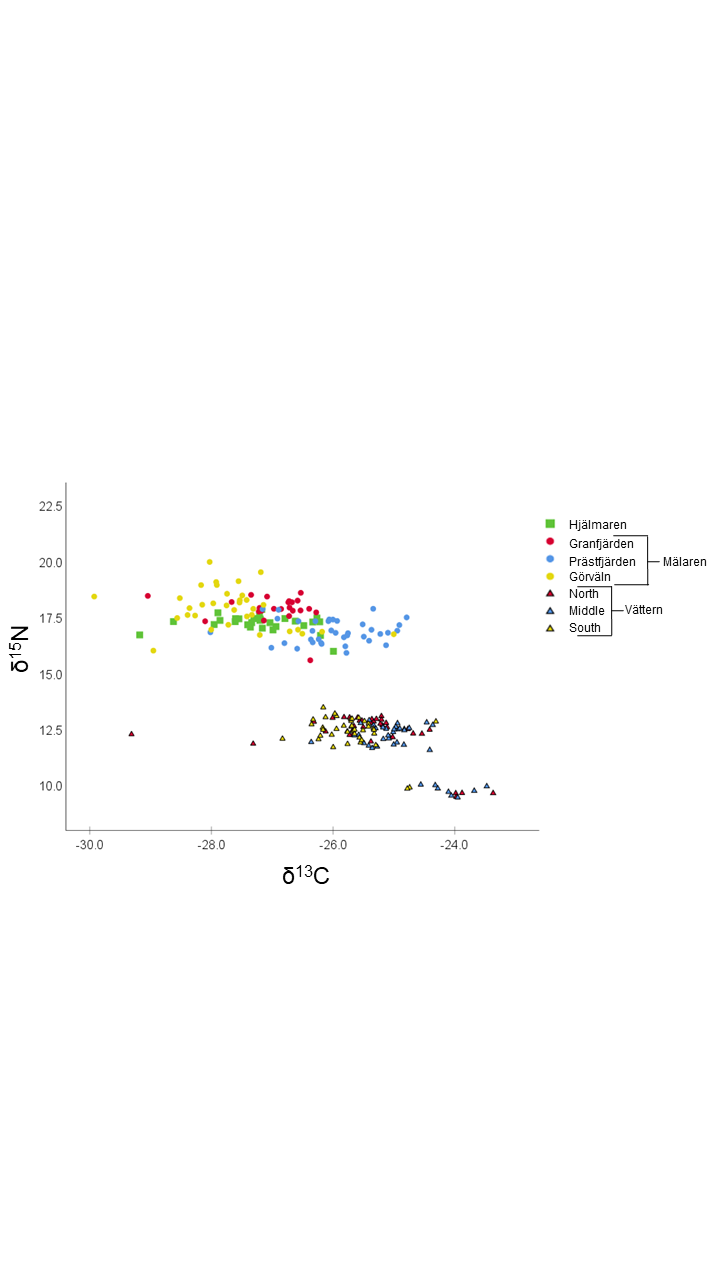

Supplement: S1 Fig — Plot includes all sizes of smelt captured during study, but does not include two outliers. Color and shape of points correspond to smelt captured in different regions and lakes, respectively. Note that all smelt with δ15N values less than 10.1 were less than 75 mm in length and removed before subsequent statistical analysis. (TIF) [file pone.0304089.s001.tif]
