# Supplementary material for: Stable isotope and fatty acid variation of a planktivorous fish among and within large lakes
Source: PLoS One. 2024 Jul 22;19(7):e0304089. doi: 10.1371/journal.pone.0304089 (PMC11262694; doi:10.1371/journal.pone.0304089)
Supplement: S1 Table — General Linear Model results evaluating smelt A) stable isotope ratios (δ13C, δ15N; all values ‰), B) ω3:ω6 fatty acid ratios, and C) fatty acids (arcsin sqrt transformed %DHA, %EPA). Region-specific model means (±SE) were evaluated at smelt total length of 97.31 mm for stable isotopes and 97.13 mm for fatty acids. Effect coefficients (±SE) are provided for intercept, length (mm), individual regions (relative to Vättern-South) and the interaction between length and region (×Length). (DOCX) [file pone.0304089.s002.docx]

Table S1. General Linear Model results evaluating smelt A) stable isotope ratios (δ^13^C, δ^15^N; all values ‰), B) ω3:ω6 fatty acid ratios, and C) fatty acids (arcsin sqrt transformed %DHA, %EPA). Region-specific model means (±SE) were evaluated at smelt total length of 97.31 mm for stable isotopes and 97.13 mm for fatty acids. Effect coefficients (±SE) are provided for intercept, length (mm), individual regions (relative to Vättern-South) and the interaction between length and region (×Length).

A)

|  |  | δ^13^C (‰) | | |  | δ^15^N (‰) | | |
| --- | --- | --- | --- | --- | --- | --- | --- | --- |
|  |  | Model mean (±SE) | Effect (±SE) | ×Length (±SE) |  | *Model mean(±SE)* | Effect (±SE) | ×Length (±SE) |
|  |  |  |  |  |  |  |  |  |
| Intercept |  |  | -24.96 ± 1.26 |  |  |  | 10.3 ± 0.86 |  |
| Length effect | |  | -0.01 ± 0.01 |  |  |  | 0.02 ± 0.01 |  |
|  |  |  |  |  |  |  |  |  |
| Hjälmaren (n = 26) | | *-26.792 ± 0.157* | -6.19 ± 1.60 | 0.05 ± 0.02 |  | *17.180 ± 0.108* | 7.81 ± 1.10 | -0.03 ± 0.01 |
| Mälaren | Granfjärden (n = 22) | *-26.992 ± 0.180* | -2.28 ± 1.67 | 0.01 ± 0.02 |  | *18.069 ± 0.124* | 7.21 ± 1.14 | -0.02 ± 0.01 |
|  | Prästfjärden (n= 36) | *-26.292 ± 0.126* | -7.06 ± 1.93 | 0.07 ± 0.02 |  | *16.814 ± 0.086* | 4.93 ± 1.32 | -0.01 ± 0.01 |
|  | Gorväln (n = 25) | *-27.717 ± 0.140* | -4.95 ± 2.03 | 0.03 ± 0.02 |  | *17.609 ± 0.096* | 6.35 ± 1.40 | -0.01 ± 0.01 |
| Vättern | North (n = 32) | *-25.711 ± 0.123* | -4.43 ± 1.89 | 0.05 ± 0.02 |  | *12.562 ± 0.084* | -0.15 ± 1.29 | 0.00 ± 0.01 |
|  | Middle (n = 31) | *-25.210 ± 0.129* | -2.19 ± 1.79 | 0.03 ± 0.02 |  | *12.229 ± 0.089* | -0.01 ± 1.23 | 0.00 ± 0.01 |
|  | South (n = 30) | *-25.825 ± 0.123* | * | * |  | *12.542 ± 0.084* | * | * |

Table S1 cont.

B)

|  |  | ω3:ω6 | | |
| --- | --- | --- | --- | --- |
|  |  | *Model mean (±SE)* | Effect (±SE) | ×Length (±SE) |
|  |  |  |  |  |
| Intercept |  |  | 2.667 ± 1.395 |  |
| Length effect | |  | 0.004 ± 0.015 |  |
|  |  |  |  |  |
| Hjälmaren (n = 28) | | *4.101 ± 0.172* | 0.304± 1.752 | 0.008 ± 0.019 |
| Mälaren | Granfjärden (n = 23) | *5.799 ± 0.198* | 6.323 ± 1.841 | -0.076 ± 0.003 |
|  | Prästfjärden (n= 38) | *5.777 ± 0.140* | 4.615 ± 0.116 | -0.013 ± 0.069 |
|  | Gorväln (n = 25) | *5.126 ± 0.157* | -2.436 ± 2.257 | 0.046 ± 0.023 |
| Vättern | North (n = 34) | *2.935 ± 0.130* | -2.799 ± 2.029 | 0.028 ± 0.021 |
|  | Middle (n = 32) | *3.546 ± 0.143* | 2.335 ± 1.988 | -0.019 ± 0.020 |
|  | South (n = 30) | *3.053 ± 0.136* | * | * |
|  |  |  |  |  |

C)

|  |  | %DHA | | |  | %EPA | | |
| --- | --- | --- | --- | --- | --- | --- | --- | --- |
|  |  | *Model mean (±SE)* | Effect (±SE) | ×Length (±SE) |  | *Model mean (±SE)* | Effect (±SE) | ×Length (±SE) |
|  |  |  |  |  |  |  |  |  |
| Intercept |  |  | 0.422 ± 0.102 |  |  |  | 0.353 ± 0.041 |  |
| Length effect | |  | 0.001 ± 0.001 |  |  |  | 0.000 ± 0.000 |  |
|  |  |  |  |  |  |  |  |  |
| Hjälmaren (n = 28) | | *0.414 ± 0.013* | -0.375 ± 0.128 | 0.003 ± 0.001 |  | *0.434 ± 0.005* | 0.037 ± 0.051 | 0.000 ± 0.001 |
| Mälaren | Granfjärden (n = 23) | *0.414 ± 0.014* | -0.137 ± 0.134 | 0.001 ± 0.001 |  | *0.475 ± 0.006* | -0.009 ± 0.054 | 0.001 ± 0.001 |
|  | Prästfjärden (n= 38) | *0.418 ± 0.010* | -0.399 ± 0.154 | 0.003 ± 0.002 |  | *0.459 ± 0.004* | 0.297 ± 0.062 | -0.002 ± 0.001 |
|  | Gorväln (n = 25) | *0.525 ± 0.011* | -0.005 ± 0.164 | 0.000 ± 0.002 |  | *0.396 ± 0.005* | 0.117 ± 0.066 | -0.001 ± 0.001 |
| Vättern | North (n = 34) | *0.485 ± 0.009* | -0.373 ± 0.148 | 0.004 ± 0.002 |  | *0.336 ± 0.004* | 0.084 ± 0.059 | -0.001 ± 0.001 |
|  | Middle (n = 32) | *0.508 ± 0.010* | -0.121 ± 0.145 | 0.001 ± 0.001 |  | *0.371 ± 0.004* | 0.089 ± 0.058 | -0.001 ± 0.001 |
|  | South (n = 30) | *0.493 ± 0.010* | * | * |  | *0.356 ± 0.004* | * | * |
