# Supplementary material for: Stable isotope and fatty acid variation of a planktivorous fish among and within large lakes
Source: PLoS One. 2024 Jul 22;19(7):e0304089. doi: 10.1371/journal.pone.0304089 (PMC11262694; doi:10.1371/journal.pone.0304089)
Supplement: S2 Table — (DOCX) [file pone.0304089.s003.docx]

Table S2: Principal component analysis (PCA) loadings and mean relative concentrations of 12 fatty acid concentrations measured in European smelt.

|  |  | **Component** | | | | | | | | | | | |
| --- | --- | --- | --- | --- | --- | --- | --- | --- | --- | --- | --- | --- | --- |
|  |  | 1 | 2 | 3 | 4 | 5 | 6 | 7 | 8 | 9 | 10 | 11 | 12 |
| **% of variance (initial eigen value):** | | 38.6% | 30.3% | 11.0% | 6.0% | 4.3% | 3.5% | 2.0% | 1.5% | 1.3% | 0.8% | 0.5% | 0.2% |
| **Fatty acids** | **Mean percent** | **Loadings** | | | | | | | | | | | |
| C14:0 | 2.7% | +0.374 | +0.868 | 0.000 | +0.096 | +0.165 | +0.028 | +0.159 | +0.040 | -0.007 | -0.179 | -0.091 | +0.050 |
| C16:0 | 20.2% | -0.230 | -0.614 | -0.258 | +0.683 | +0.121 | -0.065 | +0.049 | +0.110 | -0.018 | +0.028 | +0.023 | +0.038 |
| C16:1n7 | 3.2% | +0.740 | +0.378 | -0.140 | +0.186 | -0.418 | -0.146 | +0.054 | -0.152 | +0.173 | +0.035 | +0.019 | +0.036 |
| C18:0 | 4.8% | +0.089 | -0.873 | +0.133 | -0.024 | +0.216 | +0.280 | +0.133 | -0.204 | +0.155 | -0.053 | +0.026 | +0.010 |
| C18:1n9 | 9.3% | +0.768 | -0.245 | +0.117 | -0.256 | +0.330 | -0.389 | +0.051 | +0.008 | -0.001 | +0.054 | +0.049 | +0.050 |
| C18:1n7 | 7.5% | +0.864 | +0.316 | -0.084 | +0.035 | -0.033 | +0.269 | +0.035 | -0.047 | -0.232 | +0.022 | +0.118 | +0.026 |
| C18:2n6 | 2.9% | +0.256 | +0.836 | +0.021 | +0.145 | +0.323 | +0.177 | -0.205 | -0.017 | +0.129 | +0.135 | -0.023 | +0.007 |
| C20:4n6 | 1.9% | -0.286 | +0.174 | +0.902 | +0.063 | -0.121 | +0.081 | +0.164 | +0.121 | +0.025 | +0.085 | +0.013 | +0.028 |
| EPA; C20:5n3 | 14.7% | +0.670 | -0.610 | -0.010 | -0.163 | -0.152 | +0.165 | -0.200 | +0.215 | +0.088 | -0.060 | -0.007 | +0.062 |
| C22:5n6 | 4.9% | -0.807 | +0.514 | +0.101 | +0.024 | +0.044 | -0.079 | -0.111 | +0.013 | +0.093 | -0.129 | +0.164 | +0.013 |
| C22:5n3 | 2.2% | -0.616 | +0.273 | -0.605 | -0.291 | 0.000 | +0.122 | +0.219 | +0.125 | +0.086 | +0.088 | +0.029 | +0.030 |
| DHA; C22:6n3 | 21.1% | -0.957 | -0.077 | +0.018 | -0.086 | -0.054 | -0.003 | -0.113 | -0.166 | -0.106 | +0.030 | -0.051 | +0.109 |
